# The importance of early primary relationships in the development and psychoanalytic understanding of emptiness: connecting developmental theory with practice

**DOI:** 10.3389/fpsyt.2025.1545852

**Published:** 2025-07-04

**Authors:** Dimitrios Papadopoulos

**Affiliations:** Department of Early Years Learning and Care, University of Ioannina, Ioannina, Greece

**Keywords:** emptiness, object relations, dead mother concept, early childhood experiences, psychoanalysis, psychic development, ego defense

## Abstract

**Background:**

Emptiness is an integral component of an individual’s psychic development, characterized by the subjective human existential experience of loss and disconnection from the self, other people, and the external world. More generally, emptiness reflects the quality of internalized object relationships and the intersubjective experience of early failures in affective attunement with a responsive and regulating other.

**Aim:**

This article presents emptiness within the framework of object relations theory and incorporates Green’s concept of the “dead mother” experience. This paper aims to provide knowledge that can generate interest within the psychoanalysis community by illuminating the development and psychoanalytic understanding of emptiness, a complex condition that impacts individuals and societies.

**Case study:**

To connect theory with practice, a case study of psychoanalytical psychotherapy with an adolescent girl is presented. This clinical paradigm focuses on the subject’s states of emptiness and nothingness, which are then projected onto the therapist who is attempting to contain and transform the girl’s painful emotions.

**Conclusion:**

The object relations theory and the dead mother concept both can offer a valuable psychoanalytic perspective for understanding the intersubjective experience of emptiness.

## 1 Introduction

The subjective emotional experience of emptiness is a complicated mental state that can appear transiently or more enduringly as a feeling of being empty, as a belief that someone else is empty, or as a feeling that an individual’s overall experience precludes all other emotions (1). Although there is significant debate regarding the definition of emptiness in the field of psychoanalysis, there is no widely accepted conceptual definition. However, there is a broad theoretical consensus regarding its primary characteristics and functions. Emptiness is a subjective experience of internal deadness typically characterized by a lack of emotional vitality, diminished self-experience, and disconnection from internal or external object relations. This experience is complex and painful and lacks positive and negative feelings, desires, and fantasies (2, 3).

The expression of emptiness varies according to one’s psychic structure and function, characterizes the subjective experience of loss, and reflects the quality of internalized object relations (4). In this framework, emptiness emerges from the individual as a state of emotional deadness, in which the experience of connection with the primary object is insufficient for mental and ego development (5, 6). As the primary object represents a part of the self or enhances certain self-representations, the lack of object containment capability leads, in psychological terms, to loss of part of the self, giving rise to emotions of emptiness and mourning (7). According to Kohut (8), the long-term effects of a mother’s failure to provide an infant with regulatory self-object functions result in developmental arrest and emotional detachment. From this perspective, emptiness may result from early relational-developmental trauma, particularly when emotionally unavailable caregivers are involved. This suggests that the severe and chronic experience of emptiness, may reflect a failure in early object emotion containment and the formation of cohesive self-structures (4). This form of emptiness is often experienced as a pervasive psychic deadness leading to a more profound collapse in the ability to symbolize or mentalize (3, 9).

From an alternative perspective, emptiness might be a consequence of defensive strategies, such as repression, dissociation, or splitting, which serve to protect the psyche from excessive anxiety, unconscious fantasies, shame, or loss (4, 10). Thus, dynamic emptiness may be an internal unconscious mechanism that avoids and represses painful and overwhelming emotions (11).

Green (12) extended the object relations concept, focusing on the “dead mother” experience, whereby a child experiences a mother as physically present but emotionally dead. This experience is marked by a primary identification with an emotionally dead mother, leading to the infant’s inability to grow and remaining developmentally stuck. Green (12, p. 150) hypothesized that the infant’s subjective experience of this relationship is extremely painful and leads to a loss of both love and meaning. However, in infancy, prolonged periods of the love object’s absence result in a loss of meaning for the infant, causing a sense of uncertainty, confusion, and meaninglessness. Additionally, early signs of emptiness in an infant might appear somatically—as a physical sensation of absence, hollowness, or disintegration—and may emerge later in life as chronic detachment, identity diffusion, or a lack of emotional containment. As the child matures, the psychic void may manifest as difficulties in symbolization, affect regulation, and the capacity to mentalize both the self and others (13). In psychoanalytic terms, these challenges may give rise to a persistent sense of inner emptiness, whichcan impede the child’s ability to understand and process internal emotional states or the intentions of others as well as to give meaning to emotional experience.

In an analytic relationship, the loss of meaning and absence is demonstrated in the transference/countertransference experience. Rose (14) stresses that some patients trigger the therapist to experience a sense of emotional apathy and powerlessness in countertransference. According to Alvarez (15), this might be understood as a deficiency or dysfunction of the internal object to contain the distress and pain related to loss. Hart (16) posits that working analytically with children who have experienced the absence of a love object during early development is challenging. These children may attempt to maintain distance from the therapist to avoid any meaningful or effective interaction. In turn, the therapist may feel useless and rejected by the patient so that both individuals experience a profound sense of despair.

The individual who experiences emptiness describes a painful sense of internal emotional deadness characterized by the absence of mental representations, fantasies, and desires, as well as a poor or automatic response to conditions of relationship building or psychological connection with others. Individuals often feel hopeless about the future, cannot love or care for others, or respond to those who love and care for them. The psychoanalytic literature refers to periodic shifts from feeling depressed to a feeling of emptiness; relief from intolerable feelings of guilt and depression is often expressed as a feeling of emptiness boredom, rage, or worry, and can also dominate mental life, leading individuals to seek psychological help.

Fenichel (17) stresses that the frequent feelings of emptiness that manifest in patients with depression serve as a defense mechanism against instinctual derivatives associated with narcissistic needs and tensions that arise from the loss of love objects and injured self-esteem. In this concept, emptiness may be viewed as narcissistic and/or oral rage in response to the absence of ambivalent and narcissistic cathected objects. Emptiness is a defense mechanism against the repression of one’s forbidden instinctual impulsive needs and subsequent fantasies, thus protecting individuals from making instinctive desires and states of absence conscious experiences (18). Anna Freud (19, p. 35), in her book “The Ego and the Mechanism of Defense,” notes that patients who are blank in consciousness and feel internally empty tend to become silent in therapy, suggesting that an analogous state of emptiness may be a defense or resistance to free association. Importantly, the interruption that happens in the person’s instinctual processes during symptom development also occurs in the flow of their associations. Thus, free associations that provoke the ego’s defensive reactions are readily disregarded, blocking the verbalization of unconscious emotions (20). Consequently, psychological activity remains inhibited, leading to cognitive immobility, while experience exists without meaning and content. Ellonen-Jéquier (21) utilizes clinical material from a female psychotic patient to demonstrate that the ego can produce considerable psychic energy to construct a complex internal structure that generates a sense of emptiness or nothingness, despite its’ fragmentation and susceptibility — highlighting the paradoxical function of ego defenses in psychotic states.

In this paper, taking a developmental perspective, I present a theory that initially focuses on the emergence of object relations and continues to extend this theory with the “dead mother” experience to clarify the mental content of emptiness that accompanies its appearance and define its meaning. The following section describes some general trends in the strategies used and management of emptiness in psychodynamic practice. Next, I present a case study from my professional work with an adolescent girl who was referred for psychodynamic psychotherapy. The case study illustrates the complexity of emptiness as an experience. I discuss the girl’s defenses and my countertransference emotions within the framework of object relations and the “dead mother” concept. I also reflect both on how this conceptual framework of understanding can be used to promote a sense of aliveness in therapy as well as how it can help therapists, developmentalists, and clinicians to formulate a psychoanalytical understanding of emptiness.

## 2 Emptiness and object relations

It is generally accepted that the first object for an infant is the primary caregiver, usually the mother (22–24). Implicitly adopting Sigmund Freud’s reference to a “phylogenetic model,” Klein (24) suggests that there are object relations from the beginning of life. In contrast, Anna Freud takes a developmental approach to establishing object relations, arguing that “true” object relations develop only after the first year and replace the infant’s previous relationship with the mother, which is essentially of an oral nature (22, 23). From this perspective, object relations is inextricably intertwined with ego development and is dependent on the acquisition of cognitive structures that did not develop at the beginning of life (25). A. Freud identifies three main stages in object relations development: a) an undifferentiated or objectless stage, b) a transitional stage, and c) a stage of true object relations. The relationship between the child and mother in the first and second stages is dominated by the need for food. However, toward the end of the second stage, the infant shifts from the previous dominant experience of satisfying its own needs to an experience in which food is the source of pleasure. Thus, the infant in this second stage “loves” milk but invests emotions in the breast or the nursing bottle that is the medium via which food is offered. Finally, in the third stage, the mother becomes an internal representation regardless of whether she is satisfactory, frustrating, present, or absent, although the infant cannot accept very long absences at that age. As stated by Anna Freud (26), infants cannot differentiate between temporary absence and permanent loss. When the mother leaves, the infant does not forget her. Although the needs of the infant may be gratified by other caregivers when the mother is absent, the infant misses the mother and feels distressed. Therefore, at this stage, the infant appears to be transitioning into a more mature phase: one in which emotions of sadness and loss emerge; this is similar to the depressive position described by Klein (24). This increasing maturity is represented by the infant experiencing the primary object (mother) as a person with a mental life of her own and needs that extend beyond those of the infant. Thus, under such conditions, the state of dependency can gradually be tolerated before the infant enters a triangular relationship and experiences the Oedipal conflict (16).

From an object relations developmental perspective, the experience of object loss concerning depressive position can be related to an internalized experience of emptiness, which is an essential part of typical growth in structuring the human psyche (27). The “potential space,” as proposed by Donald Winnicott (28), highlights a transitional area between the internal and external world. This zone is characterized by an initial sense of adaptive emptiness that enables the formation of symbols and can serve as a means of negotiating reality. It also fosters the development of a cohesive sense of self, particularly when connected to an individual’s existential reflection (29).

Green (12) introduces the “dead mother” concept as a state in which the mother is emotionally unavailable, depressed, and detached from her emotions. In this condition, she becomes a dead object for the inner psychic life of the child. As the child’s need for an emotionally present object is crucial, they will attempt to enliven the “dead mother” using ineffective methods. Thus, the child will identify with the mother’s absence and unconsciously internalize it, resulting in a sense of emptiness or deadness within the child (12, p. 151). Green hypothesizes that dead mother syndrome is characterized by detachment in mother–child interactions and the absence of the love object from the child’s mind and emotions, causing the child to experience a sense of nothingness (30). These absences—which leave traces in the unconscious in the form of psychic holes—can manifest as maladaptive or pathological emptiness (9). This type of emptiness is distinguished from adaptive emptiness by the intensity and persistence of emotional numbness, which reflects a breakdown in one’s ability to think, feel, and symbolize. Furthermore, it embodies a psychic structure centered on absence, loss, and disconnection from the self and others—something is missing and expressing adherence to the inner world of an emotionally absent object (31).

Green’s (12) concept of the dead mother may present a challenge to expand conventional object relations theory, especially as articulated by Melanie Klein (24) and subsequently elaborated by Donald Winnicott (28), by incorporating a third category of internal objects—neither positive nor negative but absent/void. This illustrates that psychic structure can be influenced by the relational conflict between positive and negative object representations and a primary trauma stemming from the internalization of a non-responsive object. Consequently, a qualitatively different psychic configuration may emerge, organized around absence, unrepresented loss, psychic deadness, and non-being (16, 32). Thus, it is emphasized the essential role of being observed and loved by a responsive other in the development of stable self- and object-representations, which involve the libidinization of self and other, crucial for object constancy and subsequent symbolic capacity (33). Without this, one cannot experience a profound absence or a state of emptiness, which suggests the possibility of a remembrance or imagined presence. Recently, Clarke (34) distinguishes the experience of emptiness—characterized by a psychic void, emotional deadness, and a collapse of symbolization—from the experience of absence, which, although painful, maintains a representational link or a trace to the once-held object, allowing for grief and the possibility of preserving the object as psychically present through memory and symbolization. However, this potential diminishes if the internalized aspect is not the lost object itself but the experience of emptiness.

Using psychoanalytic observations, Bick (35) noted that not all infants seem to internalize an object that may contain their concerns and anxieties. She assumed that the infant’s skin-to-skin contact with the mother, especially between the mouth and nipple, creates the sense of a primary object. When the mother is not experienced as a psychologically available person to contain the infant’s emotions, she does not help the infant to project their emotions onto a mental object that functions as a “container” capable of receiving, welcoming, and giving meaning to the content of projective identification. Bick (36) developed the “second skin” concept to describe how the individual omnipotently creates a substitute for the contained skin, aiming to maintain its internal cohesion by replacing dependence on the object with pseudo-independence when the infant’s relationship to the object is structured in an unsafe way. In contrast to Bick, who stressed the mother’s deficit in functioning as an object containing the infant’s intolerable feelings, Meltzer et al. (37) described a structural deficit of an infant in their ability to form the image of the object as a “containment.” In their view, this inability is associated with difficulty in creating a multilevel perception of self and object, which is a prerequisite for the formation of a maternal function that contains and restrains. For example, among children with autistic-type detachments and failure to connect with an object, the child remains in a state of mental function in which there is no internal space, and no sense of separate identity has developed.

## 3 Emptiness in clinical work with children and adolescents

The psychoanalytic literature suggests that the clinical material of children with severe mental disorganization and deficiencies in ego function (e.g., children with psychosis or autism) is usually dull, repetitive, superficial, and meaningless (38). Additionally, even if information emerges, it arrives as content unrelated to mental function. In these cases, the child brings out the empty inner object that exists within, and which they must find a way to mentally integrate. This inner object is a vital part of the child’s mental life and part of their story; it cannot be erased or filled from the outside to eliminate emptiness.

In children and adolescents with early psychic organization of ego functions, in the sense that symbolic thinking has not been sufficiently developed, the psychotherapist is called upon to use their imagination and fulfill the *alpha function* for the subject to render meaningful something that is meaningless. As meaning is provided, symbols are also given until the subject can form their own symbols to transform the *beta*-elements—the primitive elements of the psyche—into *alpha-*elements. The *alpha* function, introduced by Bion (39), differs from the interpretation function, which reveals symbolic meaning. In these early mental states, no meaning has yet been created and the process of symbolism falls short. The mother’s *alpha* function performs this role, containing and transforming *beta*-elements into *alpha-*elements, and later allowing the child to introduce their own thoughts and develop their own symbols.

Accordingly, the psychoanalyst should create a state of searching for meaning and a sense of being alive through words and their vivid imagination (1, 5). This is a very difficult task because children with severe pathologies often do not perceive words as symbols, but rather as real, specific objects. I recall the example of a preschool boy with whom I worked in psychotherapy for three years. During his therapy, he was drawing only numbers, and when I asked him what he was drawing, he used to give numerical answers: “This is one, this is two…” In this case, there was nothing left but the imagination of the therapist to attribute some meaning to the child’s drawing; namely, to perform the *alpha* function, in the sense of seeking out meaning in something that is seemingly empty and lacks content.

Children and adolescents with arrested development, autistic-type tendencies, and depressive feelings usually try to turn the psychoanalyst under their omnipotent control into an autistic object—like a part of their dead self. Throughout this process, the psychoanalyst primarily feels lifeless and empty. This often takes the form of a pseudo-game in which the child dictates exactly what the psychoanalyst should say, how to behave, and how to play. Alex is a child with autism who is fascinated by numbers. We developed a “game” during our sessions in an effort to bond; in a game that he devised himself, he would ask me to count out loud painted numbers. I followed his wish, but to turn it into a game, I counted each number by pronouncing it very loudly with an intense rhythm and different sounds. Alex burst out laughing, spinning around in excitement. He asked for the game to be repeated, which eventually became dull. In such a situation, no real contact or connection with another person takes place; therefore, the child produces repetitive or superficial material that provokes mundane and meaningless interpretations or comments from the psychoanalyst (40). Under these circumstances, psychoanalysts may feel increasing anger while trying to stay alive or, worse, may be overwhelmed by a sense of utter exhaustion.

Sometimes, the child transforms themselves into an autistic object and immerses themselves in a repetitive activity to suppress unexpected emotions and interrupt the production of thought and reflection (e.g., children and adolescents, who complain of being unbearably bored and of wasting time talking about themselves or playing, seem to inhibit any form of creative mental activity). In these cases, the therapist is recommended to follow a delicate balance in their strategy: on the one hand, not to force themselves out of boredom and, on the other hand, not to become an inanimate object that has lost the ability to think and feel (41, 42).

Molinari (13) addresses how children with autism frequently participate in aimless or repetitive play that may seem empty of meaning. However, she posits that these behaviors are not meaningless but essential processes through which children attempt to understand and communicate their feelings and experiences. Observing and interpreting these behaviors can help analysts acquire insights into the child’s psychic world and the processes by which they convey feelings that may otherwise remain unarticulated or unexpressed.In transference with children, emotions and experiences from early childhood triggered by the therapeutic environment are redirected to the psychoanalyst (43). The psychoanalyst experiences the emergence of maternal emotions, which are then directed toward the child. Freud (44) stated that transference in children differs from that in adults and that complete processing of the transference (positive and negative) leading to transference neurosis cannot be achieved because the effect of the real objects of care (parents) on children’s daily lives remains crucial in childhood. Thus, the interpretation of transference is primarily formulated in psychodynamics, seeking to challenge and overcome defenses, and allowing unconscious impulses to emerge into consciousness, where they can be addressed in more adaptive ways. For example, Anna Freud’s notes from a three-year analysis of Peter Heller, a 9-year-old boy, illuminate how we understand child transference (45). The interpretation of transference is placed in the context of a psychodynamic position that combines active strategies—asking questions, containing explanations, and creating interventions (which, to some extent, may have an educational dimension)—that help children differentiate their fantasies from the real situation they are experiencing.

## 4 Clinical example: the case of Silvi

This section presents a case study followed by clinical vignettes from the first two years of psychoanalytic psychotherapy for a teenage girl. The primary objective of the following example was to acquire clinical insights by utilizing clinical vignettes of a psychotherapeutic process that described the psychodynamic functioning of the patient and the potential impact of emptiness on the interaction between the patient and the therapist (transference and countertransference phenomena). Additionally, the focus was on the therapeutic techniques required to work psychoanalytically with such states of emptiness. Furthermore, I reflect on how the object relations framework and the dead mother experience can be used to foster a sense of aliveness in psychoanalytic psychotherapy.

Silvi is a fictitious name used to protect the identity of the girl. The patient provided written informed consent for manuscript publication. To ensure complete anonymity, the specifics of the patient’s developmental and family backgrounds were not included.

### 4.1 Psychotherapeutic process

Clinical vignettes from a standard long-term psychoanalytic psychotherapy process (104 sessions), conducted at a frequency of two face-to-face sessions per week, involving an adolescent girl experiencing profound maladaptive emptiness, were extracted to elucidate the interaction between the therapist and the patient and emphasize the therapeutic principles employed by the therapist. Immediately after each therapy session, the therapist’s notes were transcribed verbatim at regular intervals to accommodate interruptions due to vacation and sickness. The notes encompassed verbatim language, emotional tone, nonverbal behavior, therapist countertransference, and changes in the therapeutic relationship. The next step was to read and analyze the material, followed by additional discussions with an external supervisor specializing in psychoanalytic theory and practice. The objective was to obtain a comprehensive understanding of the patient’s emotions and reflections, emphasizing the intersubjective experience of emptiness and object representations rather than relying on factual evidence.

The criteria employed to identify the signs of emptiness were phenomenological and psychodynamic. Phenomenologically, emptiness was identified through the patient’s experiences of inward emptiness, withdrawal tendencies, emotional flatness, a sense of nothingness, and separation from the self or others (1). Prolonged silences, the inability to symbolize and mentalize, and relational disassociation were among the indicators that emerged dynamically, frequently in response to the patient’s emotional intensity. These manifestations were analyzed in the context of the patient’s developmental history and defensive organization using psychoanalytic theoretical frameworks.

The therapy was performed in a consultation room at a busy mental health facility for children and adolescents. The psychotherapeutic intervention was performed by the principal author of this paper (DP), who is an academic with a research background and an experienced Child and Adolescent Psychotherapist trained in psychoanalytic infant observation, psychoanalytic psychotherapy, and mentalization-based treatments.

### 4.2 Background on the patient

Silvi was a 16-year-old teenage girl who sought individual psychotherapy. She experienced a low mood and diffused mental distress, and complained intensely about the emptiness, boredom, and tediousness she felt, while reporting physical pain. The pain led to an extensive examination of her biomarkers but without any pathological findings. She was underperforming at school and had several absences due to her inability to wake up in the morning to attend school on time.

Silvi was beautiful, looked older than she actually was, and took care of her appearance. Often, despite her agonizing mental pain, she maintained a smile that was fake given her lived experience and actions, which in the past included causing intense physical pain by making deep incisions in her elbow and leg with the lid of a pen. During early adolescence, she had two homosexual experiences. She was in a committed relationship with an adult male when she came for treatment.

Silvi’s parents are divorced. The relationship with her parents was dysfunctional: confrontational with the mother, albeit with occasional paradoxical dependency, and avoidant with the father.

### 4.3 Exploratory phase: assessment

Four initial diagnostic, exploratory sessions aimed to obtain an approximate assessment of her mental development, functioning, and level of organization. During these sessions, she spoke of a constant, painful feeling of emptiness that replaced all of her other emotions and made her life tedious, limited, and almost unbearable. She described various accompanying states of emptiness, such as feelings of slowness of time, embarrassment, and unnaturalness of her body movements. She admitted to a range of psychopathological symptoms, including intense sadness, episodes of self-inflicted harm, insomnia leading to sleepiness, social isolation, low learning performance, and many problems with body image. She felt unable to maintain genuine feelings toward other people and had limited social contact, apart from with her mother and sister with whom she lived. She primarily spent her days alone sleeping, reading, and listening to music while avoiding going to school. Although she presented some early talents, such as painting, drawing, and foreign languages, the pride that followed achievement was blurred as soon as her mother appeared. Silvi perceived her mother as judgmental, too present some of the time, and too absent at other times; “the root of evil,” as she would later state. Silvi’s experience of her mother’s attitude caused frustration and plunged the girl into a state of anger, pointlessness, and ultimately, mental stillness. The experience of boredom and emptiness was an attempt to protect herself from any emotional investment or process that might trigger impulses.

After the initial diagnostic phase ended, I proposed individual psychodynamic psychotherapy for the teenager twice a week, and her parents followed their own counseling work with another psychotherapist.

### 4.4 First phase of psychoanalytic psychotherapy

In the first phase of therapy, Silvi vociferously expressed feelings of emptiness, boredom, and tediousness concerning everyday life. She described the emptiness she experienced as an “internal numbness,” “a feeling of being outside myself,” and as a feeling of “there is nothing inside.” However, for several sessions it was difficult for her to describe the mental distress she felt, since she often could not find the words—and instead chose silence—to convey what she felt was happening to her. During that time, she provided two expressions that seemed to represent her mental reality: “I feel empty” and “I am deadened.” The adjectives “empty” and “deadened” that she used in reference to herself refer to deprivation and to mental emptiness, as well as to the image of an empty cavity waiting to be filled.

With Silvi, I gradually realized that I had to withstand these states of non-being, often dominated by experiences of boredom, deadness, and despair. In many consecutive sessions, she spoke about her difficulty in enjoying what was happening to her:

- SILVI: I experience the things I should be experiencing more happily as if they were an obligation (pause)… all my feelings are flat (pause)… I don’t know, I feel a monotony, a monotonous thing, I don’t understand (pause)…
- THERAPIST: From the way you describe it, you seem to feel this a lot.
- SILVI: From time to time, I experience everything 50%–50%, 50% on the side that feels happy and 50% on the side that does not feel happy.
- THERAPIST: It is like you are describing two opposite sides of yourself, the good self that is in a good mood and the other, darker self.
- SILVI: I don’t know, the way I feel it, my good self is dead … I haven’t felt pleasure in a long time, I haven’t felt it in a long time. I don’t know if I’m unconsciously putting myself into this process. Since I decided to get help, which was a very long time ago, no matter how hard I tried it myself, I couldn’t make it, and that’s why I’m here. Let’s say that my mother told me that in order to achieve something you have to find yourself first, but I could not do that, I do not know…
- THERAPIST: I wonder how you feel about your statement “I can’t find myself”?
- SILVI: I am so tired now; I am really tired (prolonged pause and silence).

A period of prolonged silence followed. It was as if she was sinking into a state of nothingness. Her eyes were somewhat closed and her body was still. In my countertransference, I imagined her dead, like a dead fetus. Concurrently, I was possessed by the feeling that I was slipping from my armchair, at risk of falling. I thought that she might have wanted to show me how she feels about being dead. I felt that she was sinking and hiding: that she had disconnected from herself and the outside world to be protected from the potential threat of a breakdown.

I pondered what had happened that dulled my capacity to think and blocked me from talking. What was the source of my disassociation? To address this challenging question, I am reminded of Bromberg (46), who posits that dissociation can occur when the patient needs the analyst to experience a sense of failure and encounter uncomfortable emotions, in the same manner that the patient’s primary caretaker failed to provide a holding environment for the child.

The predominant feeling that I would “fall” from my armchair alongside a sense of deadness, which I experienced together with the client, brought to the surface the thought of a maternal hold that was shallow and incapable of containing Silvi’s anxieties and worries, as well as the thought of a maternal object that lacked emotional availability and was unable to meet her needs. After further consideration, I realized that the dissociated element of my countertransference experience served as a starting point for Silvi to initiate a conversation within her inner psychic reality.

With a sense of concern, following some minutes of silence, I tried to find a way to communicate with Silvi without being intrusive. Then, it occurred to me that maybe she had (not) played the “Cuckoo” game; hence, I said to her,

- THERAPIST: “Cuckoo!” I’m here, where are you?
- SILVI: I was thinking about all this… (pause), though I would like to be able to do it all and have it all.
- THERAPIST: All?
- SILVI: Yes. But in the end, I have nothing… (sobbing). My mother, although not too present in my life, is a know-it-all. I don’t know why I’m crying. I would like you to understand me without me having to speak. Because that is your job.
- THERAPIST: My job is to listen carefully and understand what you are saying.
- SILVI: I don’t know, I am disappointed about coming here and talking about my mother. Even here she’s chasing me. Like I can’t get out of this relationship. And now, it is as if there is not enough air for me to breathe. I’m going to suffocate.
- THERAPIST: It’s like you feel you’re drowning in the thought that here with me you have to find what a little child finds with his mother. I wonder if you think that if you leave this suffocating relationship, I, like your mother, will take with me the air you breathe.

In the above clinical vignette, something traumatic emerged. Behind the omnipotent claim “that she wants it all,” there is an admission of failure, of what is missing and must be replaced: the absence of not just the mother but also the father and the lack of a primary relationship with those two that it comprises. I felt that when she sobbed, she needed two firm arms that should have once held her and made her feel secure in a body-to-body relationship. Roussillon (47) argues that when speech uses the body as a carrier for conveying messages, the quality of the early relationship and the feeling of security it can provide are assessed.

In the context of Roussillon’s thought, I wondered whether the tone of my voice when I repeated the phrase “*you can’t [find yourself]*” that her mother used to say to her, through my pitch and rhythm when I uttered it, plunged her into the bodily experience of frustration. This, perhaps, triggered some early entry (memory trace) of suffocation that she brought again to transference through her “disappearance.” Early memory traces associated with the mother’s voice and speech are recorded that can be unconsciously activated in the context of transference (44, 48, 49) and pervade the therapeutic relationship in analytical work.

I recognized that Silvi’s fading, alongside my own slipping from the armchair, was an unconscious request for a more “secure hold.” I felt, as Green described, that we both experienced a representation of absence and deadness in our own relationship through her own disconnection, which she had previously experienced on her own. I believe that the communication between us established a safer containing structure within the therapy, allowing for the appearance of this representation of the void. This facilitated the activation of the impulse and its orientation toward the object.

As we both had experienced that event in the present moment, she began recalling some painful memories from her early childhood. She recalled repeated childhood nightmares in which she was alone, experiencing terror, and unable to locate the path to her home after school, despite being able to see the house in the distance. Another childhood nightmare included the sudden disappearance of her mother. Although in the nightmare her mother was physically present, Silvi perceived her as a dead icon that was unable to communicate and respond to her. Predictably, as our therapeutic relationship was established, earlier memories emerged. She recalled a childhood memory from when she was approximately four- or five-years-old regarding the absent mother. In this memory, she remembers sitting alone in her mother’s armchair and looking out the window toward the area where her mother would often spend her mornings, obviously for her own pleasure. In fact, Silvi’s dreams and early memories were consistently terrifying, which triggered her to recall the conscious experience of feeling lost, bored, aimless, and empty—dead. These states, as Green says, are based on an empty representation of the absent mother, which is a wholesale identification with that deadness.

This brief review of the early phase of therapy provides only a schematic outline of some of the material relevant to diffuse emptiness. It undoubtedly sounds more organized and progressive than it actually was. However, gradually, despite many instances of stagnation of progress, the subject formed a series of organizational memories that gave meaning to seemingly meaningless and painfully empty life experiences.

### 4.5 Second phase of psychoanalytic psychotherapy

In the second phase of therapy, the nature of Silvi’s relationships with her family emerged. This particularly included the relationship with her mother, with whom she spent most of her time; with her father, whom she saw occasionally and with whom she had a distant and guiltily uncomfortable relationship; and with her mother’s partner, with whom she had a hostile relationship. Sivi’s relationship with her mother was characterized by ambivalent feelings (“I love her, but I can’t stand her. I hate her for judging me and for the fact that she has an opinion about everything”). In Silvi’s conscious life, her mother was too absent, yet sometimes experienced by the girl as over-critical. However, the maternal relationship seemed to coexist with no particular content, and a weak emotional bond, often marked by the mother’s absence and self-absorption. Silvi viewed her father’s visits as a recurring attempt to stimulate herself, to compensate for her lack of emotion with a pseudo-emotional bond, albeit incestuously colored, that left her feeling despair, partly because of what she saw as his indifferent attitude toward her, but primarily because of the insincerity of her feelings toward her father (feelings of anger and rage expressed in superficial conformity/compliance with paternal desires).

In the course of the sessions, she referred ambivalently to the mother, who at times she presented as an underrated figure, inadequate in her role (“I am afraid I will become like her, a dead person” and “she does not understand me because she is so self-absorbed”) and at times as intrusive and omnipotent (“There is distance between us since I was little,” “She wants to control me,” and “My mother is the source of evil”). In Silvi’s words, her mother was presented as disparaging and even suspicious of the father in a relationship of implicit conflict. Silvi described her father as distant but, simultaneously, protected him from her mother’s anger. Attached to the pre-Oedipal stage, teenagers cannot develop psycho-emotionally and acquire a distinct sense of self. Conversely, by identifying herself through projection, she felt that she was a link that could reunite her parents. She was the one who could calm down, make angry, and seduce an all-powerful mother, and the one who protected the father from anger and denigration.

Silvi, when faced with the demands of reality, experienced a sense of powerlessness, fearing potential failure in university entrance exams. This caused her to feel despair and guilt, which she continues to struggle with even today. In this context, moving to the limit of narcissistic rage, she became angry and attacked object relations. Her mother continued to judge Silvi and question her personal and academic choices. Her father disappointed her for not paying alimony to her mother (financial matters after divorce). Her boyfriend is about to abandon her because he must perform military service. Her anger was also directed toward the therapy. Her anger manifested through frequent aggressive actions in the therapeutic framework (cancellations, not showing up without notification, and turning up very late). This “acting out” can also be interpreted as manifestations of regression in symbolic function. It can also be linked to an attempt to deal with dependence and autonomy anxieties using earlier (pre-symbolization) mechanisms, thus opening the space for restoring the functions of the weak, narcissistic ego. By attacking the therapeutic framework, she exhibited an inability to endure her own difficult feelings and, simultaneously, through her absence, sought to protect me from her anger, as she perhaps does with her father.

## 5 Discussion

The literature review and the material of the clinical example illustrate that the experience of emptiness pertains to meeting the trapped parts of psychic activity and may be an expression of despair at reliving traumatic early relationships with an emotionally absent primary object that exists internally and needs to be embodied in the psyche. By bringing emptiness and desolation into therapy, Silvi restored the dead inner object that resided within her and needed to be integrated into her inner psychic life. Bergstein (5) states that the inner object is a vital part of the inner world, a part of the mental path, and can neither be erased nor filled in from the outside to eliminate emptiness.

In the case presented, the analytic process was challenging and took considerable effort to establish due to the subject’s severe dissociative tendencies, depressive elements, and autistic-type detachments, which were closer to dichotomy (splitting) rather than repression of subjectivation (4). Thus, the analytical relationship was developed to ensure a safe holding and foster a positive emotional experience. Following Green’s (50) suggestion, early in the therapy, I chose to listen to Silvi and hold onto her thoughts, fantasies, and words, enhancing my function as a holding space because filling the emptiness early through interpretation involves the risk of repeating intrusion of the “bad” object—in this case, her mother. She often said, “*I feel good that you are listening to me without being judgmental*.” Therapy was a safe space in which, by listening carefully, I gave a first form and presence to the “emptiness,” proof of a bond, and of continuity. Only as time passed, and after we had established the therapeutic relationship, did I choose to make some comments, as by leaving the emptiness as it was, I might have repeated the inaccessibility of the primary undesired and dead object (i.e., her mother). Green (50) also suggests that the analyst should give the patient a mental space: an experience that is neither meaningless nor saturated with meaning, always bearing in mind the multifaceted importance of meaning. In transference, Silvi communicated with me through her statement, “I feel empty,” implying that she wanted me to provide her with a positive emotional experience that would make her feel complete. Therefore, I experienced pervasive anguish about whether I could hold and contain the emptiness she brought and whether I could bring her to life—out of deadness and boredom. I experienced a painful experience of absence in Silvi’s transference to me and in my identification with her and with her maternal object. I came to the realization that the projective identification could illuminate the darkened areas of my own being. Consequently, I encountered an absence within myself that I perceived as equally authentic despite being triggered by the subject’s psyche. During therapy, Silvi experienced our analytic relationship as a continuation of the experienced ambivalence of the relationship with her mother. Thus, in transference, I alternated from the position of the idealized object to the position of the hostile object that was experienced as menacing by her vulnerable narcissistic ego.

I carefully considered interpretations by assuming a maternal function, while also maintaining and incorporating the paternal reference (47). From this perspective, the therapist can become a malleable medium (51) to address the subject’s internal desires, fantasies, and motivations, particularly the creative and dynamic parts of the self. In frozen emotional states, when feelings of boredom and deadness emerge in the analytic relationship, as I recognized with Silvi, the therapist plays an important strategic role in bearing these deadening states of anti-life and facilitating their integration through interpretation. From one perspective, therapists must surrender to the experience of numbness, deadness, and boredom to experience the emptiness and use it as a tool in the therapeutic relationship (i.e., in the transference and countertransference; 40). In contrast, when primarily withdrawn psychic states emerge, the therapist must follow a more active strategy and seek the hidden sense of being alive of their patient, in which the “absence of content” is often preferred to the presence of terrifying desires, fantasies, and traumatic memories (1). For instance, Silvi required my presence as a living object capable of delving into the depths of her deadness in which she was trapped and drawing her toward the external world. Thus, boredom, emptiness, and deadness can begin to exist both as inanimate objects found in the soul and as genuine emotions that can be explored, motivated, and restored. However, as therapy progressed and her mental capacities developed, we both confronted the withdrawn primitive object relations that were encapsulated within her psyche but had never been experienced. I realized that Silvi needed both of us to live with and experience these primitive object relations in the transference to be able to live, love, and be loved.

At the defense level, Silvi exhibited projections that were intense and massive, and her aggression was attributed to the object and to the other person, as the other person was the object of the envy. However, massive projections impoverish mental space and further restrict the already weakened functions of the ego (19). In addition, as in the clinical vignette above, disassociation was apparent. According to Winnicott (52), dissociation in an analytic relationship refers to a disconnection from reality that acts as a defense in a patient who lacks primary integration. It is the ego’s defensive attempt to organize itself against the “primitive agonies” that were experienced during infancy. Silvi’s disassociation maintained a state of silence, like a “brief sleep,” as described by Winnicott (p. 255). The dissociated silence of my patient is a significant characteristic of a regressed state, which may indicate a developmental deficiency in the preverbal stage. Indeed, this form of defense emerges when reality is too frustrating and distressing and can be understood as a defense against the risk of a breakdown (49). According to Schore (53), “disassociation” is rooted in the early phase of development that occurs in infants who are exposed on a prolonged basis and very early on to a caregiver who has a generally poor degree of coordination with them. In these cases, intense situations of long-term inadequate or poor regulation are triggered, which the body is likely to use as a lifelong regulatory process. The still-face paradigm of the mother (54) is a milestone in understanding feelings of apathy, sadness, and emptiness, the basis of which lies in sudden drops and falls of life instincts in early childhood. More specifically, infants who have been exposed from a very early age to poor or inconsistent coordination with their caregiver will develop, as a form of self-protection, lifelong cut-off mechanisms that may manifest as a message that reads “stay away, don’t connect,” thereby hampering emotional investment in primary objects.

Silvi provided a detailed account of her experiences of emptiness and deadness throughout the early sessions; however, she was not fully conscious of the source of this void within. I realized that Silvi developed a defense against awareness of maternal absence and against her own state of deadness within to protect herself from experiencing such painful emotions. I remember that during one session, I asked her to engage in mentalizing by imagining a scenario that would allow her to reflect on her feelings of emptiness and discover the root causes of her experiences. She responded to me by saying, “My mother loves me; she purchases food for me and covers the cost of my sessions.” Smith (31) presents a perspective that I completely endorse in the context of Silvi’s case: that the patient’s understanding of their own deadness is more accessible than their consciousness of maternal deadness. For example, patients may develop a defense mechanism by verifying their own perception of their mothers’ love, even if they intuitively sense that they are facing challenging emotions.

## 6 Strengths and limitations

This study connects psychoanalytic developmental theory with practice to provide an in-depth understanding of the emergence and nature of emptiness in the psychodynamic psychotherapy of an adolescent girl. It illustrates her progressive emotional expression and efforts at intersubjective connection as therapy advanced, despite the countertransference experiences being marked by feelings of deadness, disassociation, and boredom. This case provides profound insights into a “complicated real-world” treatment, ensuring that the therapeutic methods and techniques described in this study may be applicable to other real-life therapies. While case studies are essential to the psychoanalytic and psychotherapeutic literature, they are restricted by many limitations, including subjectivity, generalizability, and methodological rigor. A key limitation of this clinical paradigm is that the findings are discussed in relation to a single therapeutic process, which limits the transferability of the results to other patients or therapies. Nevertheless, the results provide insights into how therapy works in the real world (55). Additionally, the therapeutic environment is highly contextual and characterized by distinctive relational dynamics that may not apply to other therapeutic relationships.

In this case, the expression of emptiness can be significantly affected by contextual and environmental factors. For instance, in Silvi’s early childhood, Greece’s economic crisis affected numerous families, including her own, as her mother had lost employment. This may have affected Silvi’s mother’s increased likelihood of developing depression and the mother’s capacity to identify, respond effectively to, and address the child’s emotional needs. Kallinikaki (56) found that Greece’s economic crisis led to increased work hours and multiple jobs, leaving parents with limited time and resources to care for their children. This could contribute to heightened anxiety and a sense of insecurity in the parent, which, in turn, could impact the emotional care provided to the child.

Additionally, the experience and expression of emptiness in this case study may have been influenced by a pronounced stigma around sensitive issues, such as mental health and emotional challenges (57, 58). For Silvi, discussing her inner world with another individual might be a considerable barrier, even in the context of therapy, as she feared potential stigmatization or perceptions of weakness from others. It is reasonable to assume that Silvi’s decision to remain silent was an unconscious defense mechanism (e.g., against instinctual fantasies) and a conscious decision to avoid sharing her emotional vulnerability, indicating her fear of social stigmatization. Nonetheless, this case study remains a powerful tool for gaining deep insights into the psychoanalytic understanding of emptiness. It can be utilized as a case paradigm in clinical practice to enhance knowledge and deepen processes in similar situations.

Finally, this particular case focuses on the experience of emptiness in adolescence, a period of development marked by struggles with identity formation and a painful sense of internal void in terms of emerging autonomy (4, 59). In this regard, this study is limited to discussing in detail how emptiness emerges across developmental phases from infancy through adolescence to adulthood. Future studies should identify the early characteristics of emptiness experienced during childhood more clearly than the emergence of emptiness during adulthood. Future research could focus on the distinction between the therapeutic principles used to treat emptiness in children’s and adults’ psychotherapy, which are influenced by the unique needs and application of techniques pertinent to various stages of life development.

## 7 Clinical implications

This case study provides clinical evidence regarding the techniques used by a therapist working with an adolescent girl experiencing maladaptive emptiness and disconnection from the self. It illustrates how creating a therapeutic setting that offers a containing and holding environment, as described by Winnicott (52), in which the patient can begin to project and work through unformulated aspects of the self, develops a deep understanding of the therapeutic relationship during psychoanalytic psychotherapy.

Observing the therapeutic relationship is described as a core element of psychoanalytic psychotherapy for adolescents through which unconscious feelings and anxieties can be explored and understood (60). Exploring the therapeutic relationship can help uncover painful and aggressive unconscious emotions that may be directed toward the self, in line with the psychoanalytic theory of emptiness (1). In the context of treating emptiness in psychoanalytic practice, therapists should engage in a nonjudgmental and inquiring stance, creating a space where the child or adolescent feels safe to express and experience their feelings, leading to greater insight and symptom relief.

In the case presented, as a therapist, I became a capable and responsive object of containing and processing the patient’s projections, which, in turn, fostered a sense of safety and continuity, enabling Silvi to gradually explore and give meaning to dissociated or unformulated emotional states and initiate some changes as therapy advanced. Integrating André Green’s (12) concept of the “dead mother complex” into the clinical practice of emptiness, an effective therapeutic relationship involves the therapist offering a “live” and more emotionally responsive presence in reawakening the adolescent’s capacity for emotional connection, which contrasts with early experiences of emotional deadness or absence, helping restore the patients’ psychic vitality. For instance, Silvi needed my presence as a responsive object of being physically and emotionally alive to draw her out of her deadness, in which she was stuck, by providing empathic attunement, sensitivity, and reliable responsiveness, understanding them as defenses against unconscious pain, shame, or unmet needs while avoiding overly regressive interpretations (23). Recent studies suggest that transference work, when carefully tailored to the adolescent’s developmental needs and relational capacities, can foster a co-constructed meaning-making process and be an effective therapeutic tool for addressing feelings of emptiness and depression, indicating improvements in ego functioning (61, 62).

In this framework, therapists should pay close attention to transference and counter-transference dynamics, as adolescents may unconsciously re-enact early relational patterns within the therapeutic relationship, potentially idealizing, devaluing, or detaching from the therapist in ways that mirror earlier primary forms of relationships. The analyst’s function does not aim to fill the emptiness rapidly but to put vague or unarticulated emotional states into language, enabling the patient to regain the capacity for representation and symbolization in areas of mental life where only psychic deadness existed previously (31, 50).

Importantly, the work with Silvi highlighted an additional element pertinent to clinical practice: the emergence of silence, which has been extensively addressed in psychotherapy for adults, children, and adolescents (63). Silence in psychoanalytic psychotherapy may manifest as resistance, an assertion of autonomy, or a means of evaluating a therapist’s capacity for emotional intensity and ambiguity. In this case, managing silence proved difficult, particularly during the early and middle stages, as Silvi frequently had periods of silence lasting between two and four minutes. For example, in the initial phase, when the therapeutic alliance was not fully established, as Silvi exhibited states of nothingness and emotional detachment through her withdrawal, I chose to “hold” the silence—sustaining a consistent, supportive presence that provided an environment for unexpressed thoughts and emotions to manifest gradually into verbal expression. However, interpreting the silence more directly regarding the therapeutic relationship may have resulted in a more overt articulation of deeper emotional struggles during the sessions or enabled the therapist to identify challenges within the therapeutic alliance, including potentially unvoiced anger directed towards the therapist. Within this perspective, Fonagy et al. (59) propose interpreting the significance of silence in transference, aiding young people in discovering how their silence may convey latent interpersonal expectations or unconscious fears (64). Adjusting the technique carefully may be necessary when working with adolescents experiencing maladaptive emptiness. While it may be advantageous to allow for silence, an adaptation could entail reflecting the meaning of silence to the patient and explaining its application in psychotherapy (63).

## 8 Conclusion

Children who were raised by a mother who did not respond to their needs and showed no signs of being alive, “as if a dead mother,” may struggle to develop a sense of object constancy (12). The case study presented in this paper indicates that the quality of emotional connection with the primary object was at the heart of the subject’s suffering and distress. From this perspective, it is evident that this emotional connection should not be assumed in all object-subject relations. In fact, psychoanalytic theory and the clinical material both corroborate that early relationship breakdowns with the love object, who serves as the primary container, and the lack of an internalized object capable of processing and metabolizing the child’s emotions can greatly affect the child’s psychological development, leading to a “hole” in the child’s maternal representation (31).

This paper suggests that object relations theory and the dead mother concept both can offer a valuable psychoanalytic perspective for understanding the intersubjective experience of emptiness. This framework helped Silvi and me, as her therapist, avoid becoming estranged from one another. Instead, I maintained my curiosity and sense of aliveness to help my client in establishing the process of developing an attachment (16). In fact, Silvi progressively became more open to my interpretations and less repetitive in the states of emptiness she brought into therapy. Her parents reported feeling more like they existed to provide care and support for her, rather than as mere extensions of her body servicing her needs.

More importantly, psychotherapy offers Silvi an opportunity for an authentic object relationship that can welcome and metabolize the projections and progressively allow her to establish a sense of safety and stability by interacting with a good object. I believe that our analytic relationship provided Silvi with an experience by which to promote her vitality and help her to grow, even if the therapy was difficult and painful at times. This was partly influenced by both internal and external factors, including family dynamics and relationships. During therapy, I endeavored to hold and reflect Silvi’s feelings of emptiness, deadness, and occasionally resistance to her envious and destructive outbursts. I discovered that focusing on the dissociated aspect of my analytical experience worked as a catalyst for the subject to engage in a dialogue within her inner self, in a safe and growth-promoting environment.

Finally, this paper recommends that future studies should further identify more clearly the early characteristics of emptiness experienced during childhood compared to how emptiness emerges in adulthood. Future research could focus on the distinction between the therapeutical principles used to treat emptiness in children’s and adults’ psychotherapy, which is influenced by the unique needs and the application of techniques that are pertinent to various stages of life development.

## Data Availability

The original contributions presented in the study are included in the article/supplementary material. Further inquiries can be directed to the corresponding author.
